# Comparison of vaccination and booster rates and their impact on excess mortality during the COVID-19 pandemic in European countries

**DOI:** 10.3389/fimmu.2023.1151311

**Published:** 2023-07-06

**Authors:** Olga Matveeva, Svetlana A. Shabalina

**Affiliations:** ^1^ Sendai Viralytics LLC, Acton, MA, United States; ^2^ National Center for Biotechnology Information, National Library of Medicine, National Institutes of Health, Bethesda, MD, United States

**Keywords:** SARS-CoV-2, COVID-19, vaccination rate, excess mortality, booster administration, GDP, European countries

## Abstract

**Aim:**

To evaluate the effect of vaccination/booster administration dynamics on the reduction of excess mortality during COVID-19 infection waves in European countries.

**Methods:**

We selected twenty-nine countries from the OurWorldInData project database according to their population size of more than one million and the availability of information on dominant SARS-CoV-2 variants during COVID-19 infection waves. After selection, we categorized countries according to their “faster” or “slower” vaccination rates. The first category included countries that reached 60% of vaccinated residents by October 2021 and 70% by January 2022. The second or “slower” category included all other countries. In the first or “faster” category, two groups, “boosters faster’’ and “boosters slower” were created. Pearson correlation analysis, linear regression, and chi-square test for categorical data were used to identify the association between vaccination rate and excess mortality. We chose time intervals corresponding to the dominance of viral variants: Wuhan, Alpha, Delta, and Omicron BA.1/2.

**Results and discussion:**

The “faster” countries, as opposed to the “slower” ones, did better in protecting their residents from mortality during all periods of the SARS-CoV-2 pandemic and even before vaccination. Perhaps higher GDP per capita contributed to their better performance throughout the pandemic. During mass vaccination, when the Delta variant prevailed, the contrast in mortality rates between the “faster” and “slower” categories was strongest. The average excess mortality in the “slower” countries was nearly 5 times higher than in the “faster” countries, and the odds ratio (OR) was 4.9 (95% CI 4.4 to 5.4). Slower booster rates were associated with significantly higher mortality during periods dominated by Omicron BA.1 and BA.2, with an OR of 2.6 (CI 95%. 2.1 to 3.3). Among the European countries we analyzed, Denmark, Norway, and Ireland did best, with a pandemic mortality rate of 0.1% of the population or less. By comparison, Bulgaria, Serbia, and Russia had a much higher mortality rate of up to 1% of the population.

**Conclusion:**

Thus, slow vaccination and booster administration was a major factor contributing to an order of magnitude higher excess mortality in “slower” European countries compared to more rapidly immunized countries.

## Graphical Abstract

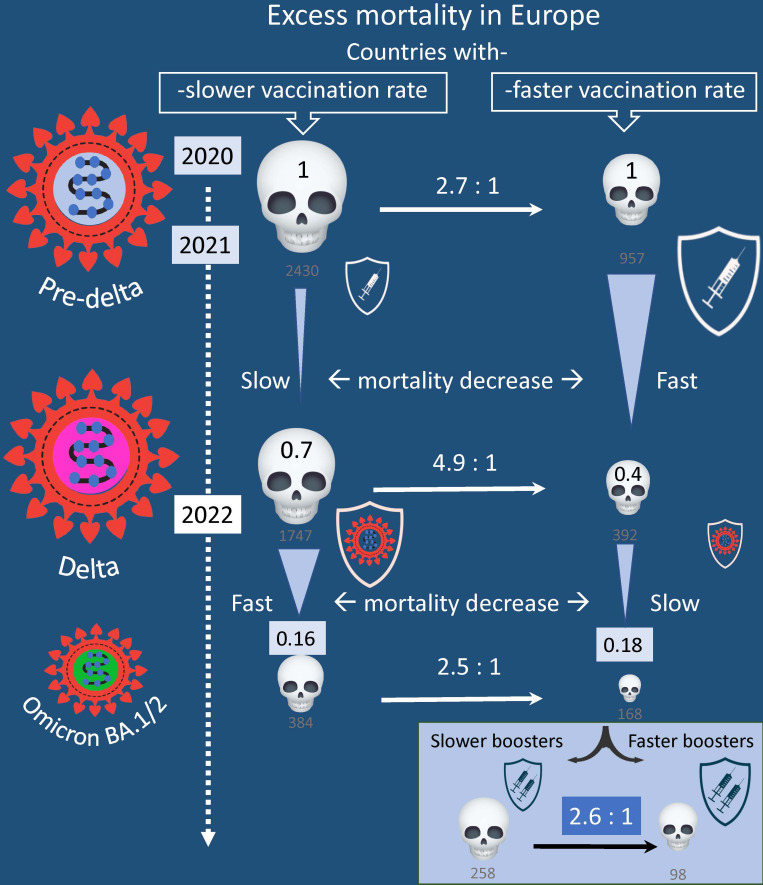

## Introduction

To deal with infectious waves caused by different variants of SARS-CoV-2, governments around the world have had to make many difficult decisions, including unpopular business closures, quarantines, and so on. An important tool in the fight against the pandemic was vaccination, which was also far from being always popular among the people of different countries and even among governments. Almost three years after the beginning of the pandemic, it is time to analyze how effective vaccination and booster administration have been during different waves of infections in various countries. One way to address this question is to compare excess mortality in different countries in which some infectious waves were synchronized, but vaccination rates varied widely. There are various vaccines that have been used to immunize populations in Europe to prevent hospitalizations and deaths from COVID-19. As shown in **Supplementary Figure 1**, approximately 70% of the doses administered in the EU in 2021 and 2022 are RNA vaccines produced by Pfizer or Moderna; the other 15% are non-replicating adenovirus vector vaccines produced by AstraZeneca and Johnson & Johnson. The origin of the remaining doses of vaccines administered in the EU and in other European countries according to the European vaccine tracker is unknown (1). Russia and China partially provided these vaccine portions for the EU and other European countries. For example, Russia supplied Hungary and Slovakia and predominantly vaccinated its own citizens with the adenovirus two-dose vector vaccine Sputnik V, which was not approved for use by WHO or EMA due to a lack of proper documentation (2, 3). China supplied several European countries with inactivated viral vaccines, which were approved by WHO (4). There are no studies comparing the efficacy of these vaccines back-to-back. However, many published studies have compared other vaccines used in Europe (5).

A meta-analysis of many studies shows similar effectiveness of the mRNA-based vaccines among themselves (6) and with the two-dose adenovirus-based vaccine produced by AstraZeneca (5, 7). Some studies show that the Sputnik V vaccine was just as effective (8). However, there is evidence that mRNA-based vaccines are more effective than the single-dose adenovirus vector-based vaccine produced by Johnson & Johnson (9). In addition, Lau ret al. (10) showed that virus-inactivated vaccines made in China had a shorter protection time compared with mRNA-based vaccines. It is also worth noting that several studies observed a significant reduction in the efficacy of all vaccines regarding protection against hospitalization or death caused by the Omicron virus variant compared with earlier virus variants (11–13).

In our analysis, we considered vaccination without analyzing which vaccine was predominantly used to vaccinate the country’s population. We believe that this is not a serious flaw in our study, since the vaccines used in Europe had similar efficacy. We have chosen to estimate excess all-cause mortality as a measure of the negative impact of the pandemic. This estimate can be made for countries that regularly publish all-cause mortality data. There are several models for estimating excess mortality that have been suggested for use during the COVID-19 pandemic (14–17). We worked with the first of these (14) for several reasons (see below), including because its estimates are available in a very user-friendly format from the OWID database, which was the source of most of the data we analyzed. At present, however, all important findings can be substantiated by employing different models.

By comparing excess mortality estimates and other characteristics of different countries, it is possible to determine which countries did better in reducing excess mortality during each infectious wave and to try to understand why. Our study is not the first one to address such questions. It is consistent with others that have analyzed international heterogeneous mortality socio-economic, regulatory, and biological consequences of the COVID-19 pandemic (15–19). In our work, however, we focused on European countries and tried to determine exactly how much vaccination contributed to reduction of mortality.

During the coronavirus pandemic, countries around the world consistently adopted various measures to reduce mortality from COVID-19. In our work, we attempted to separate the effect that these measures had before and during mass vaccination and the effect of vaccination itself on reducing excess mortality.

Both political and biological factors influence the magnitude of all-cause mortality during pandemic infection waves (15, 16, 18). All these factors can be divided into those that change slightly and those that change or may change more radically in population during a pandemic. The first category of these factors includes the age structure of the population, GDP per capita, health care structure, and so on. The second category includes factors such as 1) the rate (ratio) of lethality in SARS-CoV-2 infections, 2) COVID-19 prevention strategies that do not include vaccination, 3) vaccination rates, 4) vaccine type, 5) population accumulation of immunity from natural COVID-19 infections, 6) the length of immune protection a person receives from a vaccine or natural infection, and 7) immune escape from the virus. Each of these factors contributes to the excess number of deaths in each infectious wave. Estimating the weight of each factor in each infectious wave is not a straightforward task. It is even more difficult to assess the causal relationship between each factor and its effect on excess mortality.

Several studies have found a negative correlation between vaccination rates and excess mortality associated with COVID-19 (20–24). In theory, the mere fact that such a correlation exists cannot be proof of a causal relationship. Both high vaccination rates and low pandemic-associated mortality occur in higher-income communities or countries (25–27). This inverse relationship between income and mortality was demonstrated for the 1918-1920 influenza pandemic in Europe when individual countries were analyzed (26). A similar observation was made for the pandemic COVID-19 (2020–2021). The analysis was done at the level of individual counties and zip codes (25), and globally at the individual county level (27, 28) in the US.

High GDP per capita and high vaccination rates are factors in reducing excess pandemic mortality. This is not surprising, since countries with higher income levels have more resources to deal with pandemics. Thus, they likely had more effective protective measures before mass vaccination and had higher rates of vaccination as well. There is also a collinearity between GDP and vaccination rates; a strong correlation has been demonstrated in previously published studies as well (29). Therefore, it is important to analyze how each of the factors, namely slow rate of vaccination or low GDP per capita in the population, influenced the excess mortality rate.

In our study, we tried to assess exactly how national vaccination rates were related to the mortality peaks in Europe during the Delta wave and during the first Omicron wave. European countries are leveling off in terms of excess mortality associated with waves of infections in the second half of 2022. An increase in population immunity due to natural infections probably plays a major role in this process, especially in slower vaccinated countries. Along with this process, viral immune can escape, and antigenic drift of the virus occurs relatively quickly. Even the first Omicron variants that emerged, for example, such as B.1.1.529, were characterized by an unusually large number of mutations in their spike proteins, compared with the original strain of SARS-CoV-2 (30). As additional infections and booster immunizations occur, the level of protection of the population against the virus may increase, while at the same time it may decrease due to viral immune escape.

## Materials and methods

### Datasets of countries

**Figure 1:**
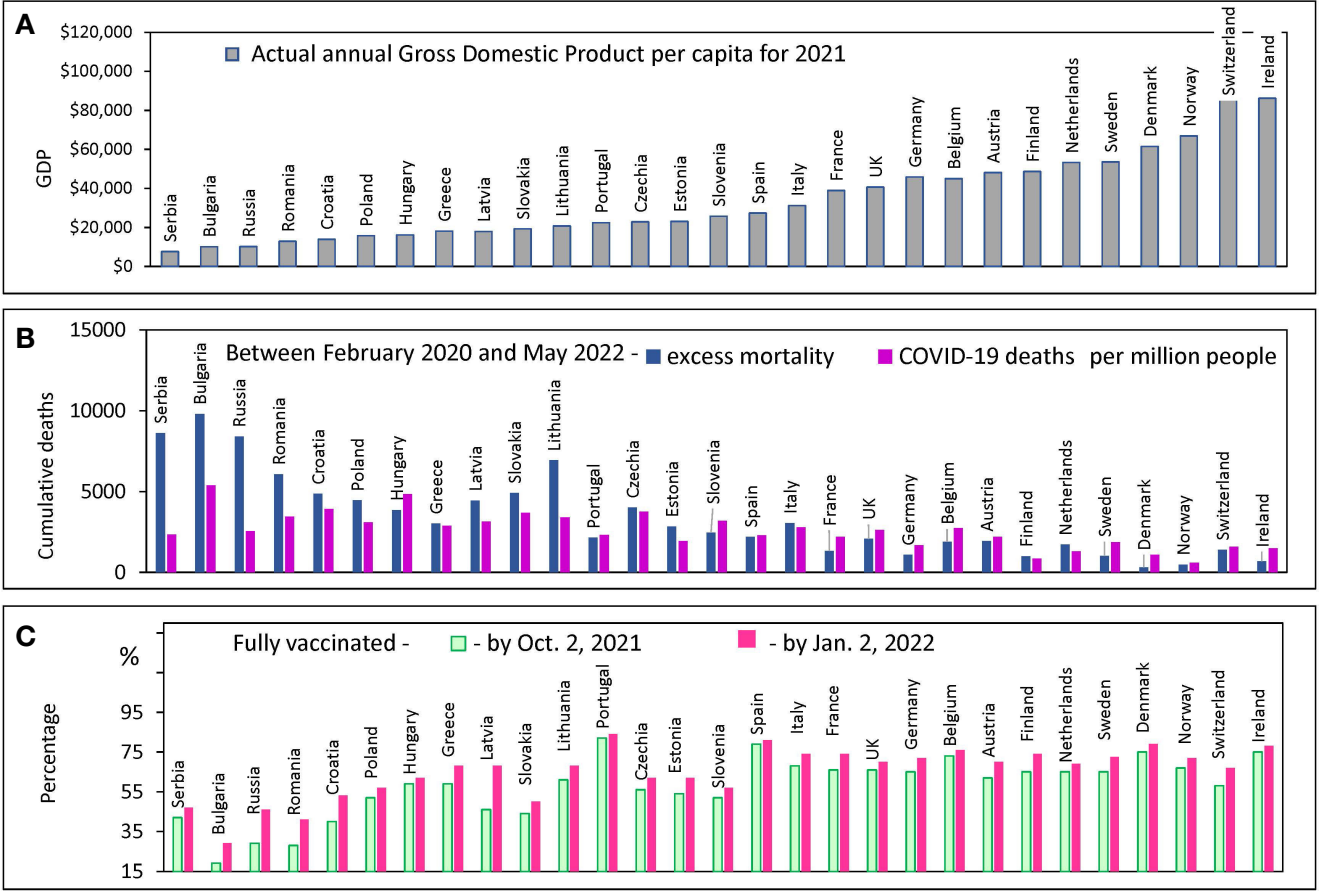
Country characteristics: GDP, Mortality and Vaccination Rates. Countries are ranked in order of increasing GDP per capita in 2021 in all panels. Standard deviations and error bars are not shown. **(A)** Actual annual GDP values from the World bank database for 29 European countries. **(B)** Excess mortality estimates, and COVID-19 confirmed deaths from the OWID database **(C)** The percentage of people who received two doses of vaccine (primary series) at certain dates.

**Figure 2:**
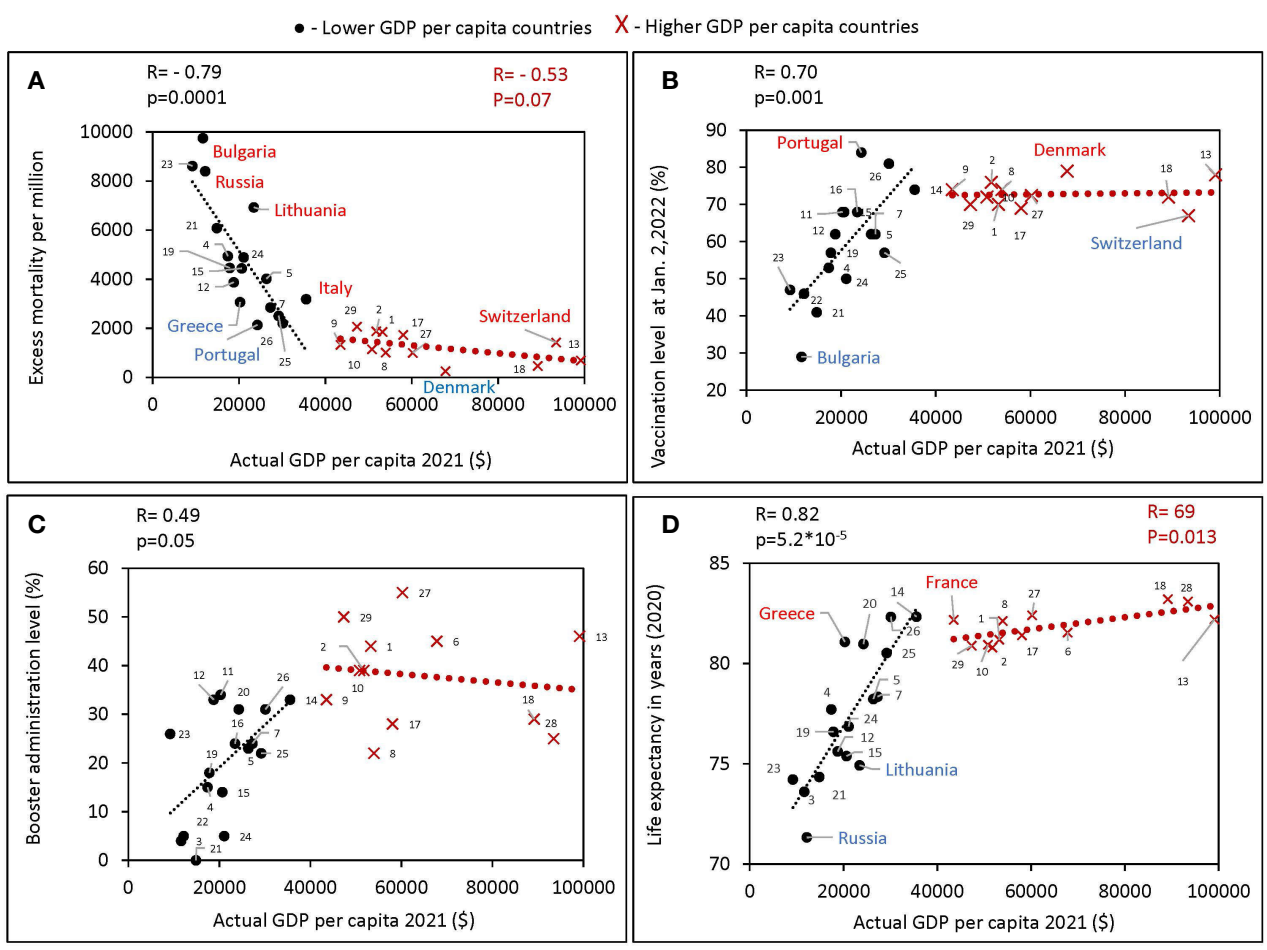
Relationship between GDPs per capita and various characteristics of European countries obtained during different periods of the pandemic. All estimates of excess mortality in the scatter plots are per million population. Higher GDP per capita countries were defined in this study as those with a GDPs equal to or greater than the threshold of $40,000, lower GDPs per capita countries defined as those with a GDPs per capita below the threshold. Countries were numbered according to the alphabetical order of their names as follows: Austria 1, Belgium 2, Bulgaria 3, Croatia 4, Czechia 5, Denmark 6, Estonia 7, Finland 8, France 9, Germany 10, Greece 11, Hungary 12, Ireland 13, Italy 14, Latvia 15, Lithuania 16, Netherlands 17, Norway 18, Poland 19, Portugal 20, Romania 21, Russia 22, Serbia 23, Slovakia 24, Slovenia 25, Spain 26, Sweden 27, Switzerland 28, United Kingdom 29. **(A)** Excess mortality during the COVID-19 pandemic from February 1, 2020, to May 30, 2022, in relation to actual GDP per capita as of 2021. **(B)** Vaccination rates, as of October 2, versus actual GDP per capita. **(C)** Boosters administration rates, as of January 2, versus actual GDP per capita. **(D)** Life expectancy versus actual GDP per capita.

We chose European countries for our work because waves of infection caused by different variants of SARS-CoV-2 were better synchronized in Europe compared to many other regions in the world. For our analysis, we selected countries that are located entirely in Europe, except for Russia, and those with a population of more than one million people. Another selection criterion was regular information updates about the coronavirus variants that were circulating in the country at any given time interval. As a result, we selected 29 countries and assigned each country a number, which we then used to refer to the country in the graphical representation of the data analysis. The countries and their assigned numbers for presentation/display in the Tables and Figures are listed in **Figure 1** and **Figure 2** legend. The names of the countries that fall into each category are shown in **Supplementary Figure 1**. Collected country characteristics for each analyzed time interval (explanation is below) were recorded in an Excel file, available as **Table 1** in the **Supplementary Material**.

### Data availability

Actual GDP per capita data were retrieved from the World Bank dataset (31). The time intervals when different variants of the coronavirus dominated were calculated from the information provided in the Our World in Data (OWID) project database.

Vaccination values were taken from the OWID database (32, 33) for each selected European country. We retrieved the percentage of fully vaccinated people for three dates: July 2, 2021, October 2, 2021, and January 2, 2022. For the countries included in our analysis, full vaccination usually means receiving a primary series: two doses for vaccines with a two-dose course and one dose for vaccines with a one-dose course. We also collected information on how many people in each country got booster doses of the vaccine by January 2, 2022. A booster dose refers to a third or fourth injection of the vaccine. The OWID database does not tell you whether the booster dose was given once or twice.

Mortality estimates were extracted from the OWID database, as numbers of confirmed COVID-19 deaths and excess deaths over various pandemic time intervals. The latter estimates were obtained using the Karlinsky-Kobak model. The model algorithm was created first by fitting a regression model for each country using historical mortality data for 2015-2019, second by using the resulting model to predict the number of deaths that can be expected in 2020-2022, and third by subtracting expected deaths from those reported for each country (14).

### Classification and analysis of data

Categorization of countries was based on their vaccination rate and countries were identified as “faster”, or “slower”. The “faster” category included countries that had reached 60% of vaccinated residents by Oct. 2, 2021, and 70% by Jan.2, 2022. The “slower” category included remaining countries that had not reached these levels of vaccination during the relevant time intervals. In the “faster” category, a subcategory with a vaccination rate of 35% achieved as of January 2, 2022, or more, was created and named “boosters fast”. We show the names of the countries that fall into each category in **Supplementary Figure 1**.

Three time periods, labeled I, II, and III, were used to estimate mortality in our analysis and in the graphical presentation of the data. The first of the periods (I) is the time interval before the start of mass vaccination from February 2020 to June 2021. The second (II) period is July 2021 to January 2022, and the third (III) is February 2022 to May 2022, both of which correspond to the time when vaccination was in full swing.

Two virus variants, namely Wuhan and Alpha, dominated the first period in sequential order, Delta dominated the second time interval, and Omicron BA.1 and BA.2 dominated the third. The precise data collection time points of the Delta/Omicron dominance switch in January or February were defined individually for each country. For some countries, this switch occurred in January and for some in February 2022. Technically, using OWID available information, we determined the date when the Delta/Omicron balance in a country approached 50% and estimated excess mortality or mortality from COVID-19 in that country after three weeks from this date. We assumed that one week after the 50% point of presence in the country, Omicron dominates. Thus, we chose the time point at which Omicron dominates, and we assumed that the mortality is mainly due to the infection associated with this strain two weeks after the corresponding date. In other words, three weeks after the date when Delta and Omicron accounted for half of the infections, we assumed that deaths occurred mainly from Omicron infection.

Data analysis, including Pearson correlation analysis, linear regression fitting, and Odds Ratio calculations were performed using Excel and in-house software. We also verified our results of categorical Chi-Square test calculation by using online tools (34, 35).

## Results

### Comparative analysis of GDP, mortality, and vaccination rates in European countries

In this study, we were most interested in the relationship between COVID-19 mortality and vaccination rates. However, when examining this aspect, we need to consider that countries differ not only in vaccination rates but also in other characteristics that may be related to population survival in a pandemic. For example, one of the most important characteristics of a country is the actual GDP per capita. This parameter can correlate strongly with the level of health care and the effectiveness of pandemic mitigation strategies, which are directly related to the reduction of excess deaths.

European countries are extremely diverse by this criterion: their GDP per capita varies by orders of magnitude: from $9,200 in Serbia to more than $90,000 in Switzerland and Ireland (**Figure 1A**). We examined how the diversity of annual income is related to the diversity of other country characteristics of interest. COVID-19 mortality rates, the excess mortality rate during a pandemic, and the vaccination rate achieved in a country at different intervals are shown in **Figures 1B, C**. All countries are displayed (X-axis) in the order of national GDP per capita growth according to the order of the countries in **Figure 1A**.

Countries with relatively low GDP per capita (less than $15K) tend to have COVID-19 mortality rates that are significantly lower than the excess all-cause mortality estimates (**Figure 1B**). In addition, these same countries with lower GDPs have relatively low vaccination rates (**Figure 1C**). The discrepancy between COVID-19 deaths and excess deaths may indicate a problem of deficiencies in disease diagnosis. A similar discrepancy was observed in several other studies published previously (17, 36). Given the problem of possible underreporting of COVID-19 deaths in some countries, we decided to analyze the data solely using excess mortality estimates.

We found a significant negative correlation between excess mortality and a country’s actual GDP per capita. The correlation is much more pronounced for poorer countries - those with GDP per capita of less than 40K (*R*=-0.79, *p*<0.0001). For richer countries, the correlation is much weaker and does not reach the 95% level of significance (**Figure 2A**).

For countries with a GDP below 40K, we found a positive correlation of GDP per capita with vaccination rates (**Figure 2B**), as well as with boosters’ administration rates (**Figure 2C**). No such correlation was observed in countries with higher GDP (**Figure 2B**). In addition, a strong positive correlation between GDP and life expectancy was found (*R*=0.82, *p*<10^-4^) for countries with GDP below 40K (**Figure 2D**). The trend was less significant for higher-income countries (*R*=0.69, *p*<0.05). Greece and France compared favorably with countries of similar income levels on average with higher life expectancy. While Russia and Lithuania stand out negatively, people in these countries lived less on average than in countries with similar income levels. During the COVID-19 pandemic, Bulgaria lost the most lives per million population and had the lowest vaccination rate among close income countries. While Denmark had the highest vaccination rate and lost the fewest lives among the European countries we analyzed.

### Estimates of excess mortality during waves of COVID-19 infection caused by different dominant virus variants

**Figure 3:**
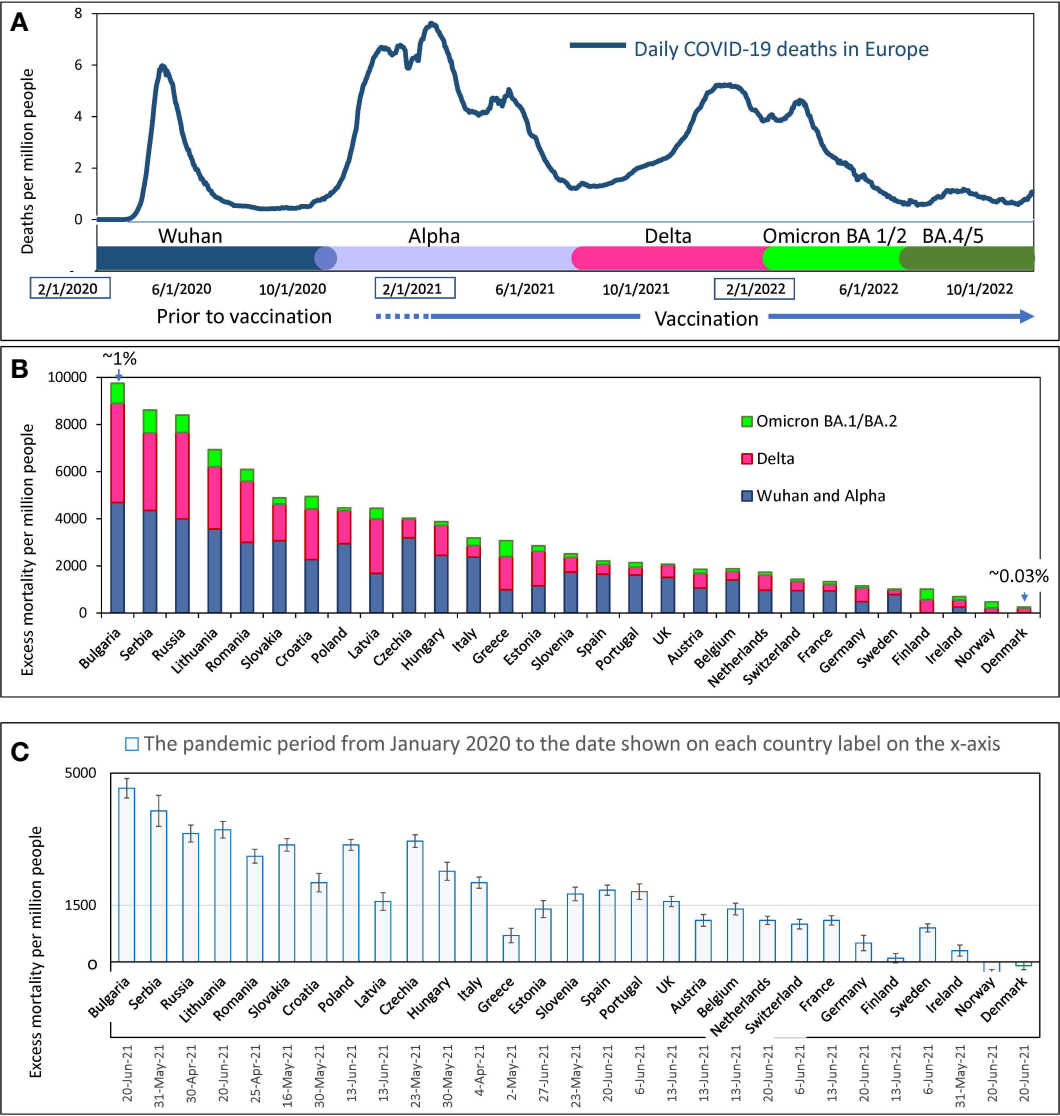
Mortality during COVID-19 pandemics in Europe **(A)** Visualization of infection waves as weekly averaged confirmed COVID-19 deaths in Europe during the pandemic through October 15, 2022. Different time intervals are highlighted in distinct colors. Information on excess mortality and dominant virus variants in each time interval was taken from the OWID database. The information is shown in more detail in the **Supplementary Figure 1**. **(B)** Excess mortality estimates for each European country for the COVID-19 pandemic period from February 2020 to May 2022. Estimates are from the OWID database and are derived from the Karlinsky-Kobak model. Each column represents three different time intervals, which are highlighted with the same colors as in “A”. **(C)** Karlinsky - Kobak model estimates of excess mortality values over the time from the beginning of the pandemic up to the summer of 2021 with the standard deviation intervals.

Coronavirus infection has spread in Europe in several waves caused by different variants of SARS-CoV-2. As reported smoothened weekly COVID-19 deaths, these waves are shown in **Figure 3A**. The first wave shows deaths caused by the ancestral Wuhan variant of the virus. The second large wave with several peaks represents deaths caused mainly by SARS-CoV-2 Alpha variant, which was dominant in Europe before the Delta variant emerged. The next large wave with two peaks shows deaths during the time interval when Delta and Omicron BA.1/2 variants dominated. Finally, the last visible comparatively small waves represent COVID-19 deaths that occurred during the time intervals when Omicron BA.5, BQ.1, and XBB 1.5 dominated.

In Europe, as noted above, the Delta-driven wave and the Omicron BA.1 wave were very close to each other and appeared as one big wave with two peaks (**Figure 3A**). However, by analyzing individual data for each country, these waves caused by different dominant virus variants could often be distinguished from each other.

The overall estimates of excess mortality, which range from a few hundred to a few thousand per million people in each country, show a contrast in the impact of the pandemic on European countries (**Figure 3B**). At the extremes of the spectrum, illustrating this contrast, are countries that have lost less than one-tenth of their residents and countries that have lost nearly one percent of their citizens’ lives. **Figure 3B** shows that countries differ in excess mortality not only throughout the entire pandemic period, but also in each time interval corresponding to each infectious wave. Thus, we showed that some countries handled the pandemic better than others, and often a country’s success in reducing excess mortality was consistent across different waves of COVID-19 infections. For example, Denmark, Norway, and Ireland handled the pandemic best in all waves, while Bulgaria, Serbia, and Russia suffered the greatest losses of life.

The excess mortality estimates presented in **Figure 3B** were calculated using the algorithms of the Karlinsky-Kobak model (14). To avoid presenting too much detail in **Figure 3B**, we have provided estimates of the standard deviation (SD) of the model calculation separately in **Figure 3C**. This analysis was done solely to reflect the confidence intervals of the calculations at the time the model description was published. The negative excess mortality values for some countries can be explained by lockdowns and quarantine, which reduced the number of car accidents. Also, social distancing and mask-wearing, reduced the prevalence of influenza and other viral infections affecting national mortality.

### Comparison of excess deaths that occurred early and late in the pandemic

COVID-19 vaccination began in late 2020, but by February 2021, only two percent of the population in Europe had been vaccinated. We compared the number of COVID-19-related casualties in European countries before and after mass vaccination began, which had a protective effect on the population and affected excess deaths. The beginning of July 2021 was chosen as the date to separate the two periods. By this date, most European countries had vaccinated over 8% but less than 40% of their fellow citizens. We present this information in more detail in **Supplementary Figure 2**.

**Figure 4:**
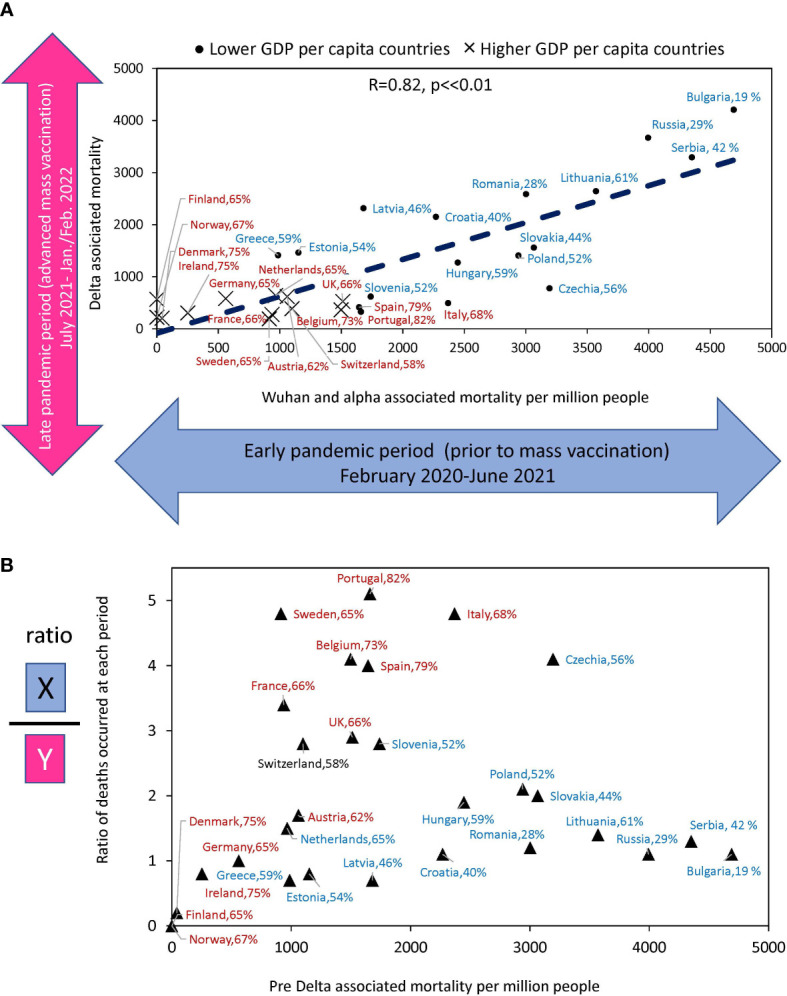
Relationship between estimates of excess deaths occurring earlier and later during the COVID-10 pandemic. **(A)** The relationship between deaths that occurred in the country during Delta domination relative to deaths whose culprits were pre-Delta virus variants (Alpha and Wuhan). **(B)** The ratio of excess death numbers that occurred in the two periods for the same countries versus to excess death numbers in the first period. The names of countries that fall into the “faster” vaccination category are shown in brown font. The names of countries tht fall into “slower” vaccination category are in blue. The percentage of the country population that got primary vaccines series by the time Delta variant arrived is shown after the name of the country.

Until July 2021, the Wuhan and Alpha virus variants prevailed in Europe, and after that month, the Delta variant, followed by Omicron. Thus, most of the excess deaths before July 2021 happened before vaccination protected people in masse. The periods we compared differ both in the level of vaccination and in the type of dominant viral variant. Wuhan and Alpha dominated for almost a year and a half and claimed a certain number of lives. Delta dominated for six months and claimed many more lives in some countries than previous variants of the virus over the same period. In other countries, though, Delta claimed far fewer lives. To address the question related to the differences in Delta associated mortality, we compared the number of deaths in each country before and after mass vaccination, when the Delta appeared. This analysis allowed us to understand how well countries protected their populations in the periods separated by July 2021 (**Figure 4**). **Figure 4A** shows that there is a significant correlation between the rates of excess mortality that occurred during the two periods of interest (*R*=0.82, *p*<<0.001).

**Figure 4B** shows the ratio of deaths between the two periods for each country relative to deaths during the first period. It illustrates how effectively countries have improved the protection of the lives of their citizens in the second period compared to the first. Portugal, Sweden, Italy, the Czech Republic, and Belgium reduced mortality by a factor of four or more, and all these countries with rapid vaccination rates. At the same time, countries with very low vaccination rates, such as Serbia. Bulgaria, Russia, and Lithuania had virtually no reduction in losses. Thus, some countries, after experiencing high mortality in the first period, radically changed their trajectory and protected their populations much better in the second period, when faced with a Delta variant of the virus. The results indicated that those with higher vaccination rates reduced mortality in the Delta wave more than those with lower vaccination rates.

### Correlation analysis of excess mortality estimates versus vaccination rates

**Figure 5:**
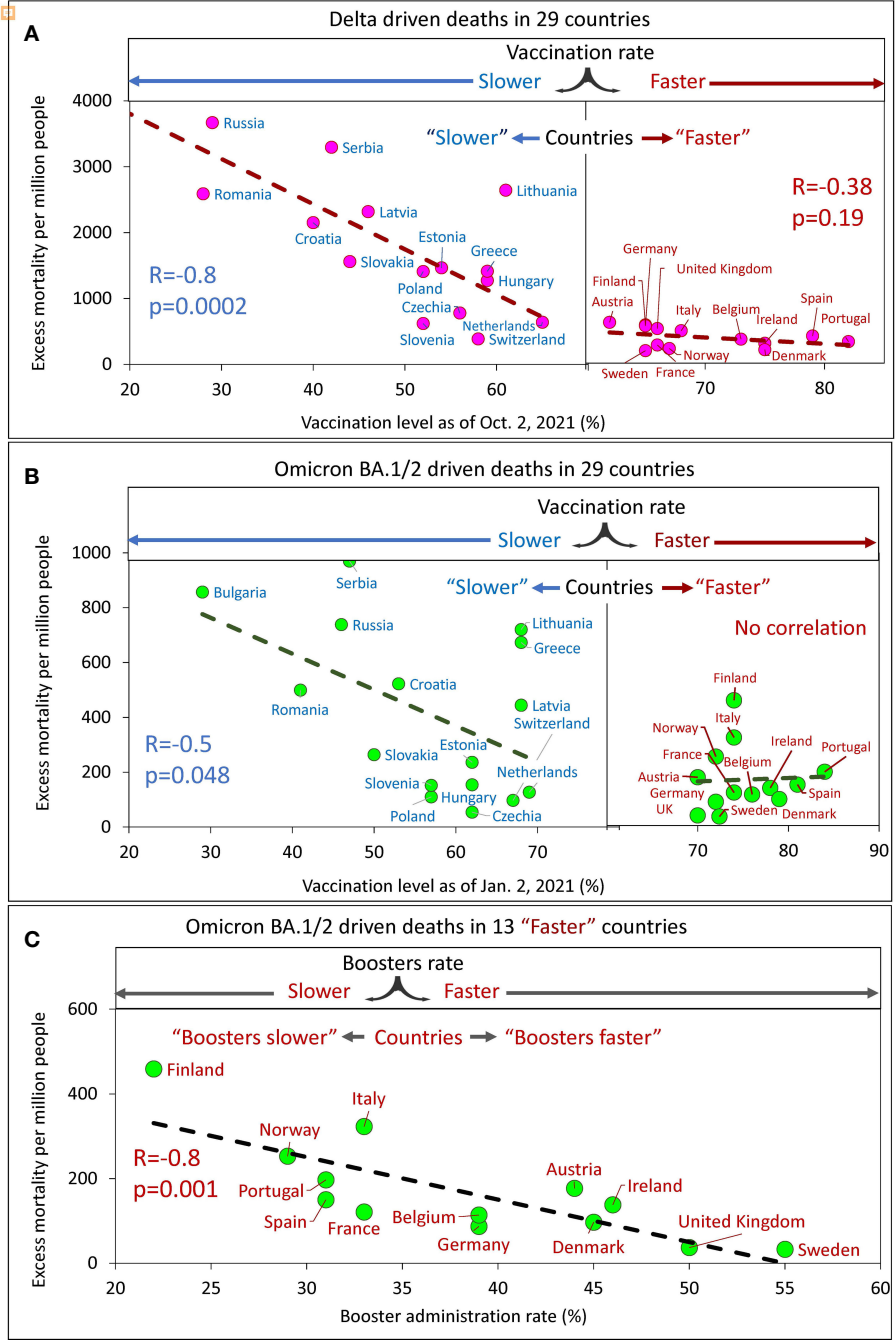
Excess mortality and vaccination rates in European countries estimated for different time intervals. **(A)** Delta associated excess mortality by country *vs*. vaccination rates. **(B)** Omicron BA.1/2 associated excess mortality by country *vs*. vaccination rates. **(C)** Omicron BA.1/2 associated excess mortality by country *vs*. boosters’ administration rate in the “faster” vaccination rate country category.

In the context of factors that may affect COVID-19-related mortality, we analyzed the effect of vaccination/boosters rates on estimated excess mortality over time. For each country, an excess mortality value was presented versus vaccination or booster administration rates achieved at specific time points (**Figure 5**).

Analysis of the data showed that a higher vaccination rate in a country corresponded to a lower excess mortality rate. By sampling the data and linear interpolation between points, we found that the best linear trend of decreasing mortality as the vaccinated population in a country increased was in countries with vaccination rates below a certain threshold. This threshold is somewhere between 60 and 70% of the vaccinated population in the country. Thus, we found that for countries with vaccination rates below the threshold; the correlation is very strong and significant, while for countries with vaccination rates above the threshold, the correlation is weak, marginal (**Figure 5A**) or virtually not-detectable (**Figure 5B**). Low excess mortality, which was not significantly different between countries, was observed in all countries with vaccination rates above the threshold. This observation was accurate for the Delta-dominated period (**Figure 5A**) and for the Omicron BA.1/2 dominated period as well (**Figure 5B**). Such an analysis of the data allows us to divide more accurately the countries into two categories: those that vaccinated faster and those that vaccinated slower. The countries with higher vaccination rates reached some threshold level and equaled them in terms of excess mortality rates.

At the same time, for the Omicron BA.1 and BA.2 period, we found that in countries in the “faster” vaccination category, while mortality was practically independent of vaccination rate, it depended on booster rate (**Figure 5C**).

In summary, a significant negative linear relationship between excess mortality and vaccination rates was found only for “slower” countries. This was true both for the Delta dominant period (*R*=-0.8, *p*<0.01) and for the Omicron BA.1 and BA.2 dominant period (*R*=-0.5, *p*=0.045). We did not find significant relationships for countries in the “faster” category, likely because all countries in the “faster” category have relatively low mortality rates. However, an inverse linear relationship between mortality and the rate of booster administration was found for the “faster” countries during the Omicron-dominated period. The greater the percentage of the population that received booster doses, the lower the mortality rate (*R*=-0.8, *p*<<0.01).

Far more people died from COVID-19 in countries that were slow to vaccinate their populations compared to countries that did it faster. The same can be said for the speed at which countries provided additional doses of booster vaccine - the higher the speed, the fewer deaths.

### Age characteristics and excess mortality

**Figure 6:**
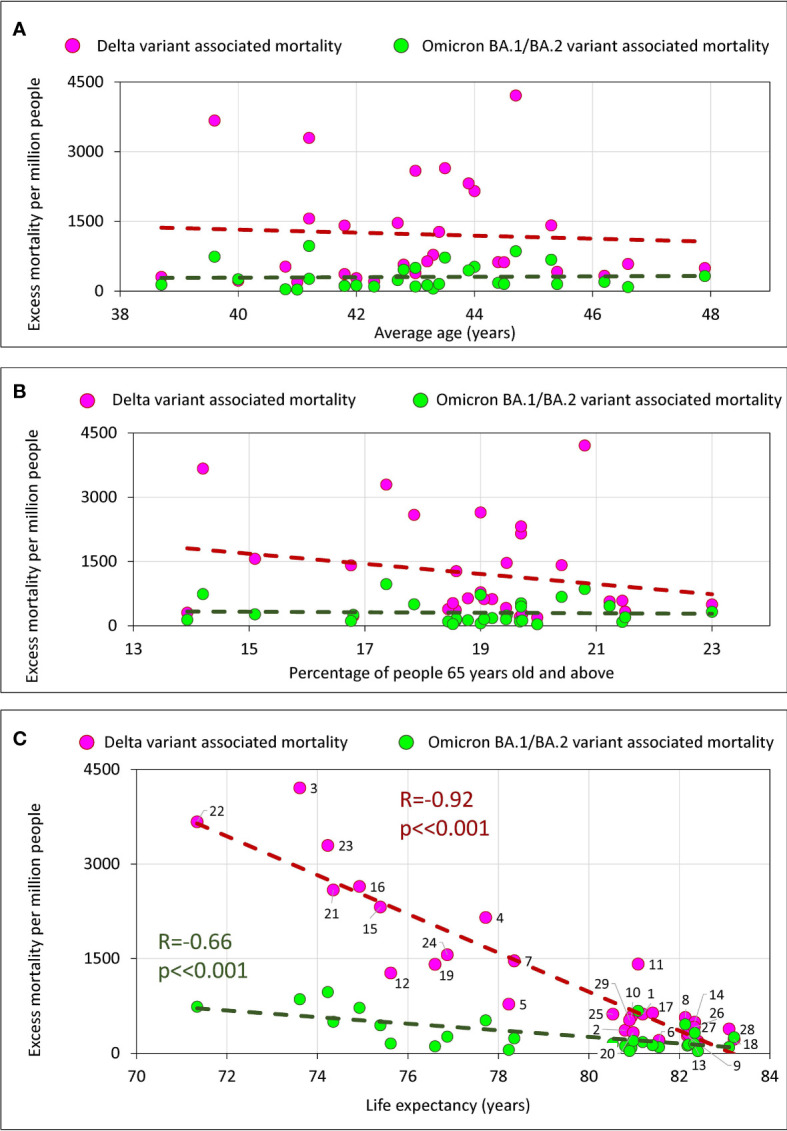
Excess mortality and the average age of the population in the country, the percentage of the elderly, and life expectancy in the country. **(A)** Excess mortality and the average age of the population in the country. **(B)** Excess mortality and the percentage of the elderly population (65+) in the country. **(C)** Relationship between excess mortality and life expectancy.

To examine how a country’s age characteristics were related to excess mortality we chose three features: the average age of the country’s population, the percentage of the elderly population (65+), and life expectancy. From the three characteristics listed, only life expectancy of the population is the most related to the quality of life and health care. The average age and the percentage of the elderly population (65+) are less dependent as they are strongly influenced by the percentage of young people in the country. The results of a correlation analysis of these three characteristics with excess mortality, which correspond to infection waves caused by Delta and Omicron BA.1/BA.2 virus variants, are presented in **Figure 6**.

There is no significant correlation of excess mortality in a country with the average age of a population (**Figure 6A**) or with the percentage of the elderly population (**Figure 6B**). However, we do see a strong significant negative correlation between life expectancy and excess mortality (**Figure 6C**). The longer a life expectancy in a country, the lower the pandemic excess mortality for both the Delta wave and the Omicron wave.

We performed a similar correlation analysis using the same parameters separately for the categories of countries that vaccinated their populations faster and slower (**Supplementary Figure 3**). In this analysis, we found a trend with marginal significance for set of countries that vaccinated more rapidly, showing that the higher the average national age, the higher the excess mortality. However, we found no correlation between excess mortality in a country and the percentage of the elderly population, either when analyzing all countries together or when analyzing the categories of countries in which the populations were vaccinated at different rates (**Supplementary Figure 3**).

### Analysis of GDP per capita and vaccination rate using regression models

In trying to establish a causal relationship between vaccination rates and reductions in excess mortality, we tried to understand what factors other than vaccination contribute to saving lives of the nation’s citizens. Considering that some of these factors may be related to GDP, this is not a straightforward task. We showed that the level of a country’s GDP itself is significantly correlated with the level of vaccination (**Figure 2B**), and this result is consistent with previously published data (19, 29).

**Figure 7:**
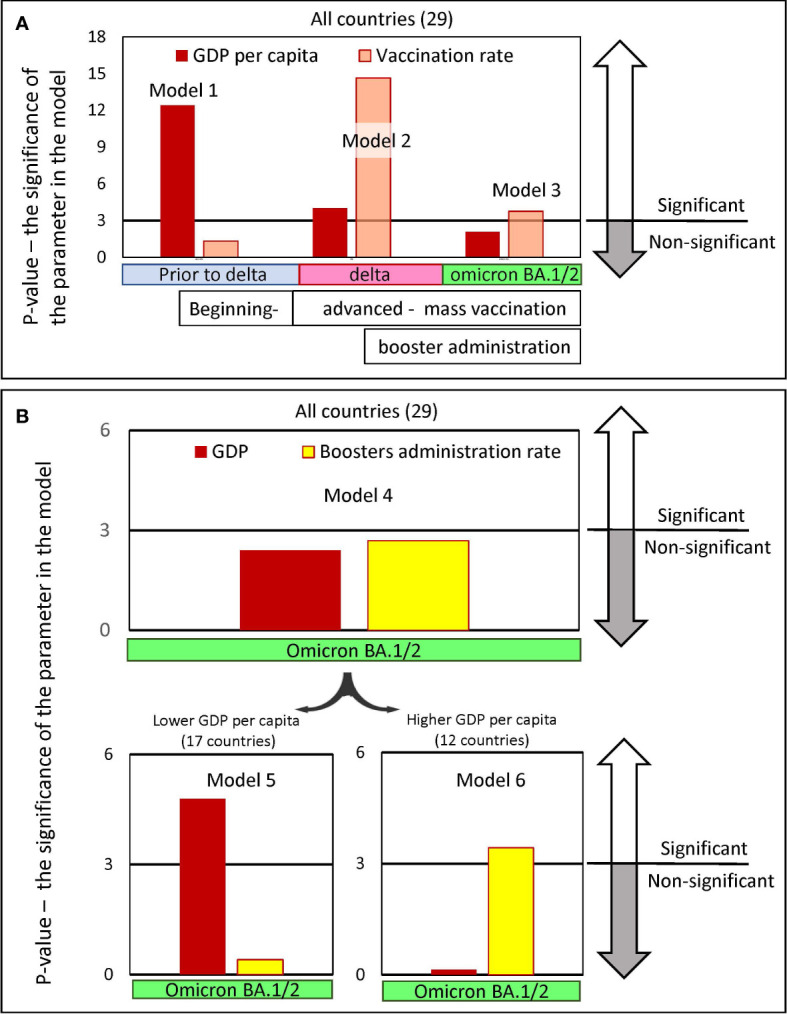
Significance levels of input parameters in linear regression models predicting pandemic excess mortality. The regression models were created to predict excess mortality. Mortality was chosen as the dependent variable, while GDP per capita and the vaccine immunization rate of the country’s population were chosen as the independent variables. The models’ outputs are presented in **Table 2** in **Supplementary Material**. Each figure column represents a negative natural logarithmic value of the significance level of the corresponding model input parameter. The pandemic periods, for which we built models, differed in the dominance of the viral variant. Wuhan and Alpha variants dominated Europe, before July 2021, afterwards the Delta variant dominated until January/February 2022 and later until May 2022 Omicron BA.½. **(A)** Input parameters are GDP and vaccination rates in the country, achieved in the respective time periods. Models 1-3 were created for the set of all 29 countries. **(B)** Input parameters are GDP and the level of boosters in the country reached by a certain time. Model 4 was created for the set of all 29 countries, and models 5 and 6 were created only for countries subsets with GDP per capita below or above 40K, respectively. There are 17 countries in the first subset and 12 in the second.

To address this issue, a set of linear regression models with variable inputs was created. In all proposed models, mortality was used as the dependent variable, while GDP per capita and country vaccination or booster rates were treated as independent variables. The results of the linear regression analysis are described in detail in **Table 2** in **Supplementary Material** and are presented in **Figure 7** as columns showing the level of significance assigned to each parameter in that each model.

We found that to predict excess mortality, depending on the time interval when the deaths occur, either the levels of GDP or the vaccination rate or both may be significant as input parameters (**Figure 7A**). Model 1, which considers mainly COVID-19 mortality in the period before mass vaccination and before the dominance of the Delta variant, assigned a level of significance only to the GDP parameter. In contrast, Model 2, which considers mortality that occurred when the vaccination process was advanced and the Delta variant dominated, assigned significance levels to both input parameters: namely, GDP and vaccination. Finally, model 3, which analyzes mortality during the period of advanced vaccination, when the Omicron variant dominates, assigns significance levels only to the vaccination rate. It should be noted that the vaccination parameter appears to be much more significant in Model 2 compared to Model 3.

Thus, summarizing the results, we can conclude that the factors affecting mortality reduction related to the country’s income played the greatest role before mass vaccination began, but the least afterward. We can also conclude that the level of vaccination played a greater role in preventing Delta-induced deaths compared to Omicron-induced deaths.

We examined models in which GDP and the level of boosters achieved in a country by the time the Omicron virus variant emerged were considered as input parameters (Model 4-6, **Figure 7B**). The analysis of all countries in Model 4 showed that neither GDP nor booster administration are significant input parameters that can dramatically affect mortality during Omicron dominance. However, we found that GDP and vaccination rate can affect excess mortality in the Omicron wave when categories of countries with lower and higher GDP per capita are analyzed separately.

During the Omicron wave, the GDP parameter is most significant in determining high mortality for relatively low-income countries (**Figure 7B**, Model 5). In contrast, for countries with higher income, GDP is not significant, but the rate of booster administration is highly significant in the model (**Figure 7B**, Model 6). Because the level of booster administration in low-income countries is not quite high, it is difficult to expect an important role for this parameter in Model 5. This analysis demonstrated that the rate of booster administration was important for preventing deaths in high-income countries during the period when Omicron BA.1/BA.2 dominated. Thus, we showed that when mixing countries with different levels of GDP, models could not produce reliable results.

### Comparison of COVID-19-related mortality in “faster” and “slower” countries during the dominance of different SARS-CoV-2 variants

Four epidemic waves caused by Wuhan, Alpha, Delta, and Omicron viruses BA.1, as well as BA.2, resulted in different excess mortality rates in different countries, as shown in **Figure 3B**. We were interested in knowing how the magnitude of excess mortality in different periods of the pandemic depended on which category a country was in, namely whether it was “faster” or “slower” in its vaccination.

**Figure 8:**
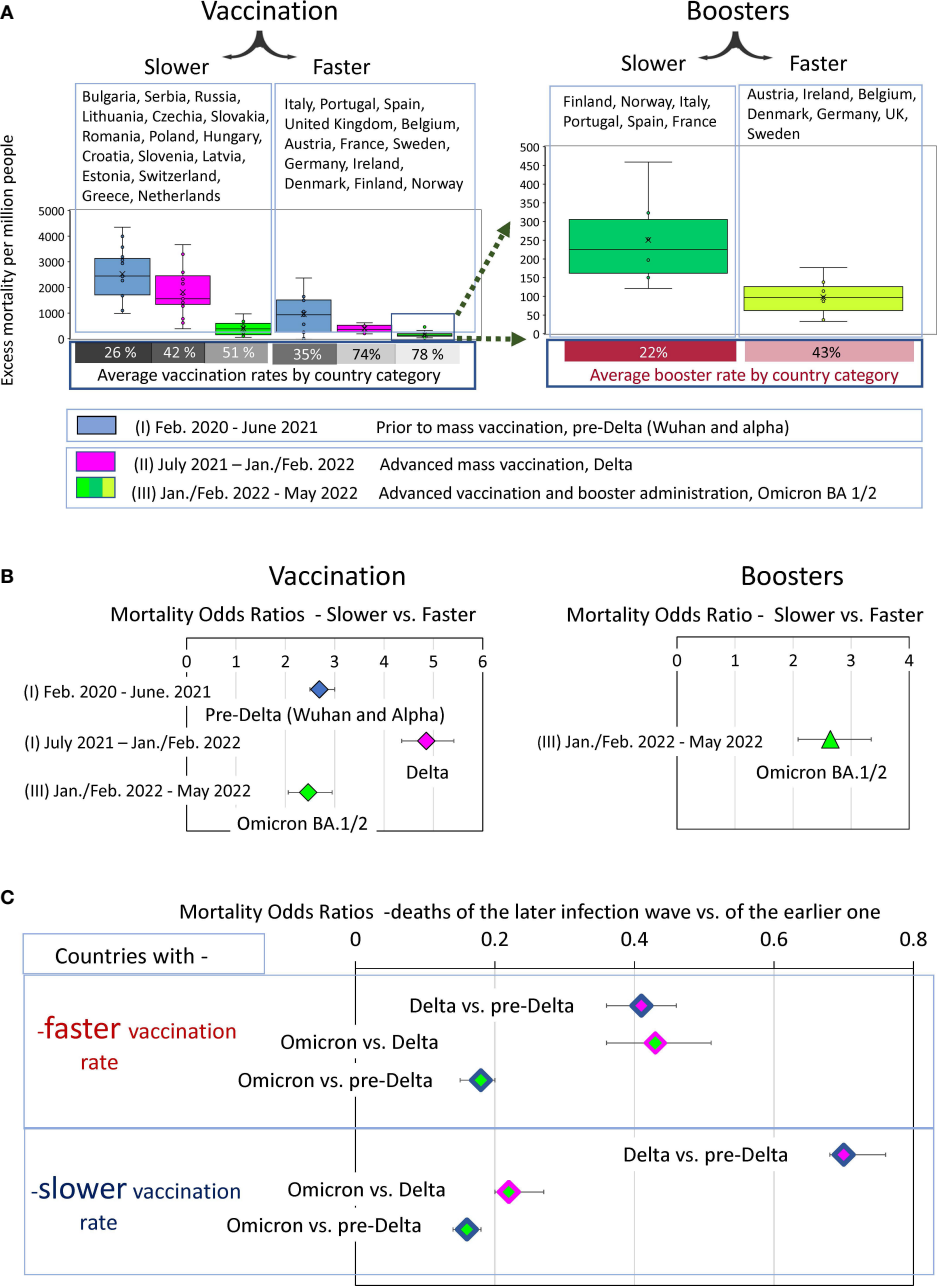
Analysis of COVID-19 associated excess deaths averaged for country categories. **(A)** Distribution of excess mortality values in two categories of countries at three different time intervals of the pandemic, visualized as boxplots. **(B)** Odds ratio of dying in each time interval depending on the country category. **(C)** Odds ratio of dying in later infection waves versus the previous infection waves depending on the country category.

To answer this question, the distributions of excess mortality values were visualized as box plots for both categories of countries for the three intervals of the pandemic (**Figure 8A**). The first interval (I) included the time prior to mass vaccination where viral variants Wuhan and Alpha (pre-Delta strains) dominated. The second (II) and third (III) periods correspond to the time when vaccination was in full swing, where Delta dominated in the second interval and Omicron BA 1/BA.2. in the third. We then assessed the mean values in each data category and their ratios. A comparison of these averages and their ratios showed how mortality during each pandemic period was related to the category of country in which the individual lived (**Figure 8B**). A low probability of dying during a pandemic was associated with better protection against all-cause mortality provided by the countries in the corresponding category.

The “faster” countries, compared to the “slower” ones, were much better at protecting their residents throughout the pandemic. However, the difference in protection effectiveness depended on the time interval. For example, the odds of dying in the first pre-Delta time interval were nearly three times higher for residents of countries that failed to provide prompt vaccination in the future compared with those that did (OR 2.7 (95% CI 2.5-3)). The contrast in the odds of dying between these same categories of countries became much more pronounced, reaching almost fivefold in the second time interval, when vaccination was advanced, and the Delta variant dominated (OR 4.9 (95% CI 4.4-5.4)). However, the contrast halved in the third time interval, when people died mostly from the Omicron variant to an OR of 2.5 (95% CI 2.1- 2.9). For the same wave of Omicron infections, there was more than a twofold difference in mortality for the “boosters slower” versus “boosters faster” subcategory with an OR of 2.6 (95% CI2.1-3.3).

### Analysis of excess mortality during successive waves of COVID-19 infections

To evaluate the dynamics of mortality-change in transitions between different COVID-19 pandemic waves, we measured odds ratios between mortality in the late and early infectious waves for “faster” and “slower” vaccinated countries separately. We visualized the excess mortality distributions as box plots and estimated the odds ratios of mortality between Delta and pre-Delta, or Omicron and Delta waves and (**Figures 8A, B**).

The comparative analysis of the odds ratio in **Figure 8C** demonstrated that the first transition was much more pronounced for the “faster” countries compared to the “slower”. Mortality during the Delta wave was less than half of pre-Delta mortality in the “faster” category (OR 0.4 (95% CI 0.36-0.46)) and more than half in the “slower” category (OR 0.7 (95% CI 0.68-0.76). In other words, during the transition to the Delta wave, mortality rates declined strongly in the “faster” countries and weakly in the “slower” ones. In contrast, the transition between Delta and Omicron waves was more pronounced for the “slower” countries. In this category, Omicron-associated mortality was only one fifth of that of the Delta wave (OR 0.22 (95% CI 0.2-0.25)). In contrast, in the “faster” category, Omicron accounted for just under half of the Delta deaths (0.43 (95% CI 0.36-0.53)). Nevertheless, the odds ratios of pre-Delta versus Omicron deaths represent similar values in both categories of countries (**Figure 8C**).

Thus, we can summarize that the degree of mortality reduction during the transition from infection waves caused by pre-Delta virus variants to the Omicron variant was independent of the rate of vaccination in the countries. However, the trajectory of this decrease depended on this rate. We have seen a sharp decline between pre-Delta and Delta mortality for the “faster” countries. At the same time, we observed a strong mortality reduction between Delta and Omicron waves for the “slower” countries. The difference in trajectories led to the major difference in mortality between the two categories of countries during the Delta infection wave. The ability of rapid vaccination to save lives was best exemplified by the Delta wave, and the ability of rapid booster administration to save lives was best exemplified by the first Omicron wave.

## Discussion

### Estimates of excess mortality versus COVID-19 confirmed deaths

The underreporting of COVID-19 deaths in some countries is a well-known phenomenon, which was thoroughly discussed in previously published studies (14). The global worldwide estimate is 18 million excess deaths between early 2020 and the end of 2021, while reported COVID-19 deaths over the same period are about 6 million, three times less (37).There are several reasons for the discrepancy between reported and excess COVID-19 deaths. For example, medical reporting systems may not list COVID-19 as a cause of death if a person has not been tested for SARS-CoV-2, and thus deaths caused by the virus may be missed in official counts in countries with low testing rates. Early in the pandemic, before widespread testing, many COVID-19 deaths among the elderly were not related to the disease, causing a significant underreporting in some countries (16).

Thus, our study found that in some countries there is a disparity between excess mortality and deaths directly related to COVID-19 which is not unexpected. The existence of this discrepancy, and the fact that it occurs primarily in countries with relatively low GDP per capita, is consistent with what has already been found and published (14, 17). In summary, all these findings underscore the fact that excess mortality is a more reliable indicator of pandemic deaths than COVID-19 direct mortality, which has been diagnosed as a direct result of COVID-19.

### Low GDP, low vaccination rates and high pandemic-associated mortality

Our study showed that countries with low GDP per capita have higher mortality rates. A negative correlation between excess mortality and GDP per capita has been observed before. It has been seen for Spanish flu (26) and for COVID-19 pandemic (27, 28). Even at the single-country level, the excess mortality associated with COVID-19 is inversely correlated with the average family income that existed in the area of residence (25). Thus, our observations are consistent with those found earlier in published studies. Not surprisingly, richer countries have more resources to deal with the pandemic-induced problems, so they do a better job of reducing excess mortality overall.

Also not surprisingly, our analysis demonstrated a positive correlation between GDPs per capita and vaccination rates. The data showed that countries with relatively low incomes were slower to vaccinate their citizens and ended up with lower vaccinated populations. Similar observations have been described in detail in the research publications (19, 29). It has also previously been observed that low vaccination rates in countries coincide with underreporting of COVID-19 mortality (18). In this context, the results of our analysis of European countries, which show a discrepancy between COVID-19 and excess mortality as well as low rates of vaccination in countries with low GDP, are consistent with previous findings.

### Age, life expectancy and excess mortality

COVID-19 is more dangerous for elderly people, and deaths occur primarily in the older population (38). In our analysis, however, we found only a weak relationship between the average age of people in a country and excess mortality (**Figure 6A**). The correlation did not reach statistical significance and was detected only in those countries where vaccination was faster (**Supplementary Figure 3**, lower left panel). We also didn’t find any correlation between percentage 65+ people in a country and excess mortality (**Figure 6B**; **Supplementary Figure 3**, middle panels.) This may indicate that the level of medical care and vaccination rate play a greater role in saving lives than the average age of the population or percentage of elderly population in a country. This conclusion is also supported by the strong negative correlation between life expectancy and excess mortality (**Figure 6C**). The higher the life expectancy, the fewer lives the country lost during the pandemic. This is true for both the Delta wave and the Omicron wave.

### Finding a causal relationship between COVID-19 pandemic mortality and rates of vaccination and booster

Analysis of pandemic mortality across countries allows us to examine the effectiveness of vaccines and boosters during different periods of the pandemic when different variants of the coronavirus were prevalent. In addition, such analyses allow an assessment of how well vaccination worked against the background of immunity triggered by natural infections. The significant and strong negative correlations we observed between national vaccination rates and excess mortality seemed to answer the question of vaccination effectiveness in reducing COVID-19 mortality in an obvious way. However, a closer analysis of the data showed that other factors, namely public health effectiveness, quality of healthcare, and the efficacy of pandemic mitigation strategies, must also be considered to assess the impact of vaccination on saving lives. Isolation of the impact of these factors is not a straightforward task. They are all linked and act synergistically.

In our work, we analyzed the data to distinguish the contribution of these listed factors from the vaccination rate factor. In doing so, we assumed that annual actual GDP per capita largely determines the amount of funding available to national governments to implement all life-saving strategies, including those not related to vaccination rates.

Our analysis shows that “faster” countries that achieved higher vaccination rates had lower pre-vaccination excess mortality compared to countries with low vaccination rates. However, the difference between “faster” and “slower” countries became much more pronounced when mass vaccination was in full swing. From this we conclude that although countries differed in the effectiveness of COVID-19 mortality control measures before vaccination, vaccination made these differences much more pronounced. Thus, vaccines greatly improved the effectiveness of pandemic control measures.

In this study, we found the existence of a certain threshold level of vaccination, namely 60-70% of the country’s population. Countries that reach this threshold quickly differ little in their mortality rates in comparison to the slower vaccinating countries, where the difference was significant. However, during Omicron dominance, despite the threshold reached, the countries that reached it, still differed in terms of excess mortality, and the magnitude of this excess mortality correlated inversely with the level of booster vaccinations. The immuno-compromising characteristics of Omicron likely contributed to diminished protective effect of the vaccination.

Our work pointed to the great importance of rapid administration of boosters before January 2022. Mortality in countries with rapid booster administration was significantly lower than in countries with the same per capita GDP, the same vaccination rate, but lower booster rates. The results of our data analysis are consistent with the observation that additional booster doses of both mRNA- and adenovirus-vector-based vaccines significantly increase the protective efficacy of vaccine against severe disease (13).

### The transition from one infectious wave to another and the associated change in mortality in “faster” and “slower” countries

In our work, we have shown that the overall rate of COVID-19 related deaths varies across countries and depends on many factors, including the level of vaccination. However, even before vaccination, countries in the category where the population would subsequently be vaccinated more quickly had lower excess mortality rates. Apparently, this is due to the fact that these countries have on average, higher GDP per capita and, accordingly, more capacity to mitigate the epidemic consequences. At the same time, during the period of mass vaccination, and especially during the period of dominance of the Delta variant, the ability to reduce mortality increased sharply in the category of countries that were rapidly vaccinating their population. The contrast in terms of excess mortality between rapidly or slowly vaccinating countries became particularly strong. Accordingly, the inverse correlation between the number of vaccinated people in the country and excess mortality became particularly pronounced during the Delta infection wave. This correlation weakened in the next infectious wave, namely, the Omicron-dominated wave. In fact, the difference between fast and slow vaccinating countries became the same as it was before mass vaccination began. There are several explanations for this phenomenon. First, the time has passed since the vaccination and the immune vaccine defense has weakened. Second, Omicron has a more pronounced ability to resist immune defenses, and finally, immune defenses increased after natural infections in countries with low vaccination rates.

It is worth noting that booster vaccinations played a hugely positive role in reducing mortality during the Omicron BA.1/BA.2 dominated period. In countries with comparable levels of GDP per capita and similar rates of primary series vaccination, excess mortality was largely determined by the level of booster vaccination administration during the Omicron-dominated period. This observation tells us that even though Omicron antigenically is very different from the ancestral SARS-CoV-2 strain, for which the vaccines were produced, booster vaccination effectively prevented excess mortality in the first waves of Omicron, caused by BA.1 and BA.2 virus variants.

Our work revealed an interesting pattern, namely that the degree of reduction in excess mortality when comparing the pre-Delta to Omicron waves was independent of the rate of vaccination. In **Figure 7C**, we showed that Omicron mortality is about one-sixth that of pre-Delta mortality in both the “faster” and “slower” categories. However, mortality decreases equally only in the transition from the pre-Delta wave to the Omicron one. As for the intermediate transitions, they are very different in the two categories of countries. The most pronounced reduction in excess mortality occurs in the transition to Delta in the category of countries with rapid vaccination and in the transition to the Omicron wave in the category of countries with slower vaccination rates.

A possible explanation is that rapidly vaccinated countries developed immunity faster, mostly in the pre-Delta pandemic phase, and slow-vaccinated countries developed hybrid (vaccine plus disease) immunity in later phases, likely during the Delta wave of infection. Thus, immunity, which saves from death, developed more rapidly in some countries, mainly due to vaccination, and more slowly in others, because of the hybrid influence of vaccine administration and natural infections. Despite all this, however, the minimum number of deaths in the first wave of Omicron was in countries with the highest rates of booster vaccination. Our results demonstrated that booster protection can still have a significant impact and reduce excess mortality despite high levels of immunity in the populations of countries in both categories.

### Hybrid immunity

Excess mortality during the Omicron BA.1/BA.2 waves is approximately one-sixth that of pre-Delta mortality in both “fast” and “slow” countries (**Figure 7C**). We believe that this finding indicates that by the time the Omicron variant appeared, the general immunity of the population had already developed and could be comparable between countries from both categories. The protective shield of established immunity significantly reduced the number of deaths during the Omicron wave compared to previous periods. It has been shown that prior SARS-CoV-2 infection and booster vaccinations provide strong protection from Omicron caused ICU admission or deaths (39–41). Therefore, we can assume that the immunity protective shield was formed as a result of vaccination or COVID-19 disease and, importantly, sometimes as a combination of both, in the same person, namely as a results of hybrid immunity. The latter is particularly important and has recently been shown to be able to protect more effectively than natural immunity acquired by people from vaccine alone or disease alone (42–44). It is likely that the percentage of people with hybrid immunity increased significantly in subsequent waves of Omicron variants and thereby strengthened population immunity. The existence and maintenance of population immunity can probably explain, at least in part, the absence of large infectious waves in the second half of 2022 in most countries.

Many studies have compared immunity from vaccines, natural and breakthrough infections and have shown that hybrid immunity protects better against SARS-CoV-2 variants compared to immunity from vaccine alone or COVID-19 alone (42–44). When vaccinating previously infected individuals, just one dose of vaccine enhances both B- and T-cell response to various variants of the virus (45). In individuals previously infected with COVID-19, vaccination induces the production of cross-variant neutralizing antibodies (46).

In some countries, especially in China, the formation of hybrid immunity, in particular its component derived from natural immunity against the disease, has been severely delayed. This is primarily due to a three-year policy of rigorous testing, contact tracing combined with strict quarantines and even lockdowns, commonly referred to as the “zero covid” policy. This policy was introduced in China in January 2020 and continued until December 2022 (10, 47).

Vaccine immunity is gradually lost, and the rate of loss of protective effectiveness depends on the type of vaccine. China used types of vaccines based on an inactivated virus. A comparison of these vaccines and mRNA-based vaccines used in many European countries and Hong Kong during the Omicron BA.2 outbreak in March 2022 showed that vaccines based on an inactivated virus protected people for a shorter period of time than mRNA based vaccines (10).

In addition, the percentage of the booster-vaccinated population among the elderly, who are particularly at risk for fatal or severe COVID-19, was relatively small in China. Some scientists familiar with the epidemiological situation in China have advised urgently increasing this percentage (47). Thus, the abrupt cancellation of China’s zero-covid policy in late 2022 led to a dramatic outbreak of disease, leading to a significant increase in hospitalizations in December and January. Already in the first week of January, the number of hospitalizations, according to the WHO, doubled and mortality rates also rose sharply (48).

Natural immunity to COVID-19 in Hong Kong in January 2023 was probably better than in mainland China because the island survived a severe outbreak of Omicron BA.2 in March 2022. Some experts estimated that about 40% of Hong Kong’s population was infected during the outbreak (OurworldInData (32),). It is worth noting that the vaccination situation in Hong Kong is better than in mainland China because almost half of the population received the mRNA vaccine, which has a longer protection period than the inactivated virus-based vaccines used in China (10). Considering these data, it can be assumed that the situation in mainland China can only be worse than in Hong Kong. The daily mortality rate on the mainland seems to be reaching a higher level than on the island, where there were nine deaths per million people in January 2023. However, using Hong Kong data, even conservative estimates for China’s population of 1.4 billion predict a daily death rate of 12600.

Hybrid immunity has probably developed faster in countries that do not have such radical policies as China. It has been shown that regardless of the vaccine used, hybrid immunity induces a stronger humoral response than vaccination (44). Hybrid immunity may also provide greater protection than immunity induced by vaccination alone against the Omicron variant (49). We assume that some infectious background of continuing circulating SARS-CoV-2 variants along with booster vaccinations will maintain hybrid immunity in most countries going forward. As a result, we can expect that in 2023 most of the world, and especially the European countries considered in this study, will avoid major infectious outbreaks such as the one that occurred in China in January of this year.

## Conclusions

- Slow vaccination and slow booster administration have been associated with high excess mortality from COVID-19 in European countries. In contrast, high vaccination rates provided robust protection against virus-associated mortality. Vaccine protection peaked in the Delta wave but became weaker in the Omicron wave.
- However, additional booster vaccination was very effective in preventing excess mortality caused by the Omicron BA.1/BA.2 infectious wave.
- The main trend found in this study was that the European countries that vaccinated their populations faster were mostly the same countries that had higher GDP per capita. They also provided better protection against COVID-19-related deaths even before vaccination campaigns began.
- Although a small number of countries protected their populations from COVID-19 deaths poorly before vaccination campaigns began, they did much better afterwards by ensuring fast vaccination of their citizens.
- The excess mortality during the COVID-19 pandemic correlates not only with a county’s vaccination rate, but also with its per capita GDP. The latter parameter likely reflects and is related to the quality of healthcare in the country, the availability of mass COVID-19 testing, and funding for other pandemic mitigation strategies.

## Data availability statement

The original contributions presented in the study are included in the article/**Supplementary Material**. Further inquiries can be directed to the corresponding authors.

## Author contributions

OM - Data curation, Formal analysis, Investigation, Figures design, Writing. SS - Conceptualization, Methodology, Writing. All authors contributed to the article and approved the submitted version.

## Supplementary material

Supplementary Figure 1 Share of people that completed the primary series vaccination at a given date and distribution of different vaccine types in EU.

Supplementary Figure 2 SARS-CoV-2 sequences share by variant.

Supplementary Figure 3 Relationships between national excess mortality in countries and their age characteristics. The correlation coefficient values corresponding to statistical significance (p<0.05) are shown in white on a light brown background for the Delta period and in dark green for the Omicron period.
