# Supplementary material for: Comparison of vaccination and booster rates and their impact on excess mortality during the COVID-19 pandemic in European countries
Source: Front Immunol. 2023 Jul 6;14:1151311. doi: 10.3389/fimmu.2023.1151311 (PMC10357837; doi:10.3389/fimmu.2023.1151311)

All countries were categorized according to their "faster" or "slower" vaccination rates. The first category included countries that reached 60% of vaccinated residents by October 2021 and 70% by January 2022. The second or "slower" category included all other countries.

February 1, 2021

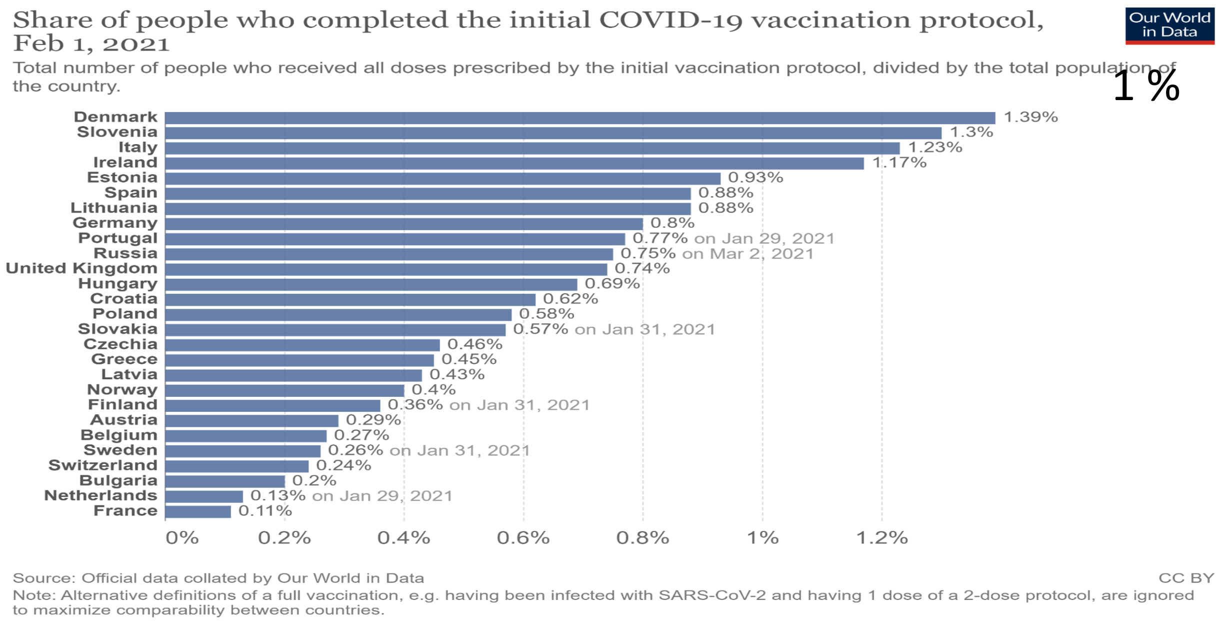

June 1, 2021

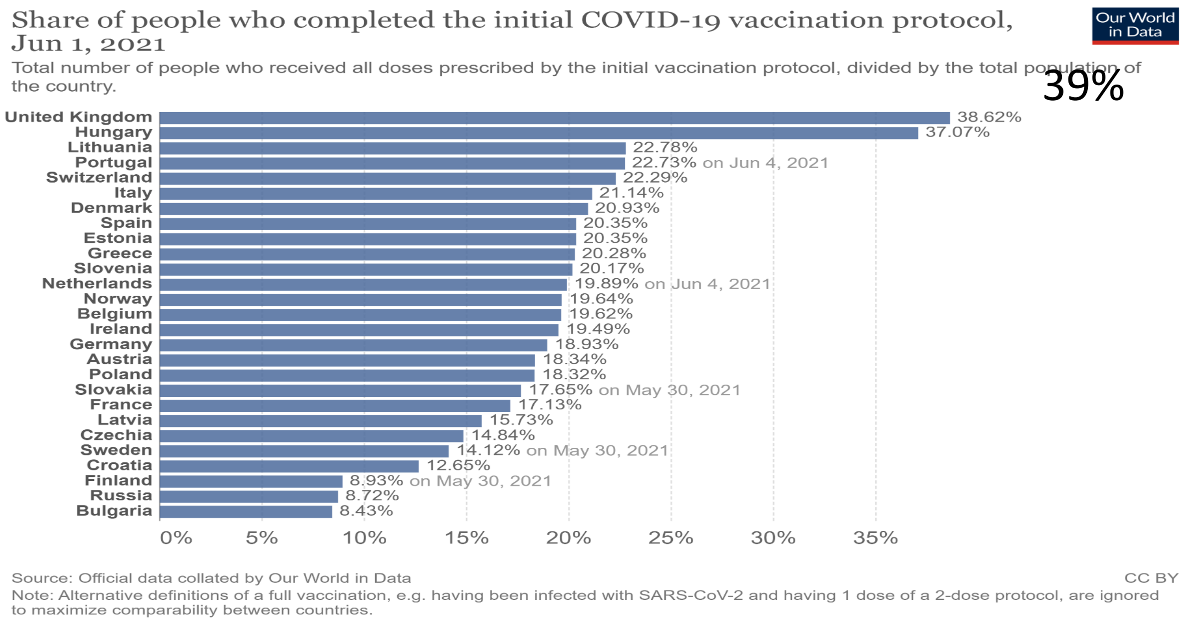

July 2, 2021

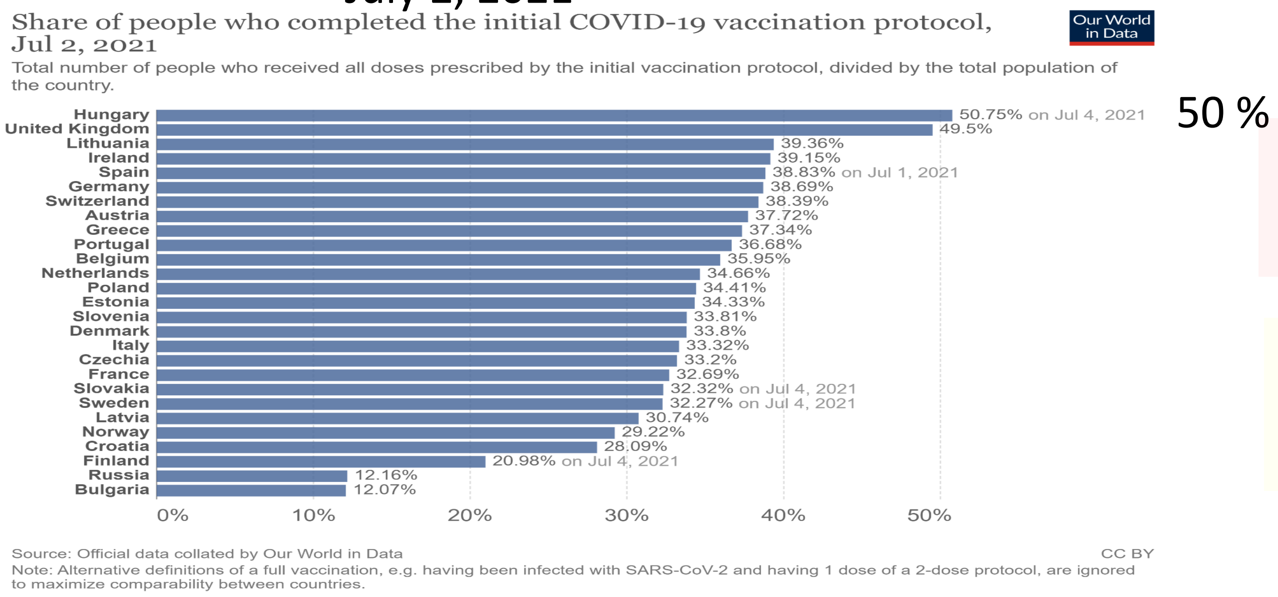

October 2, 2021

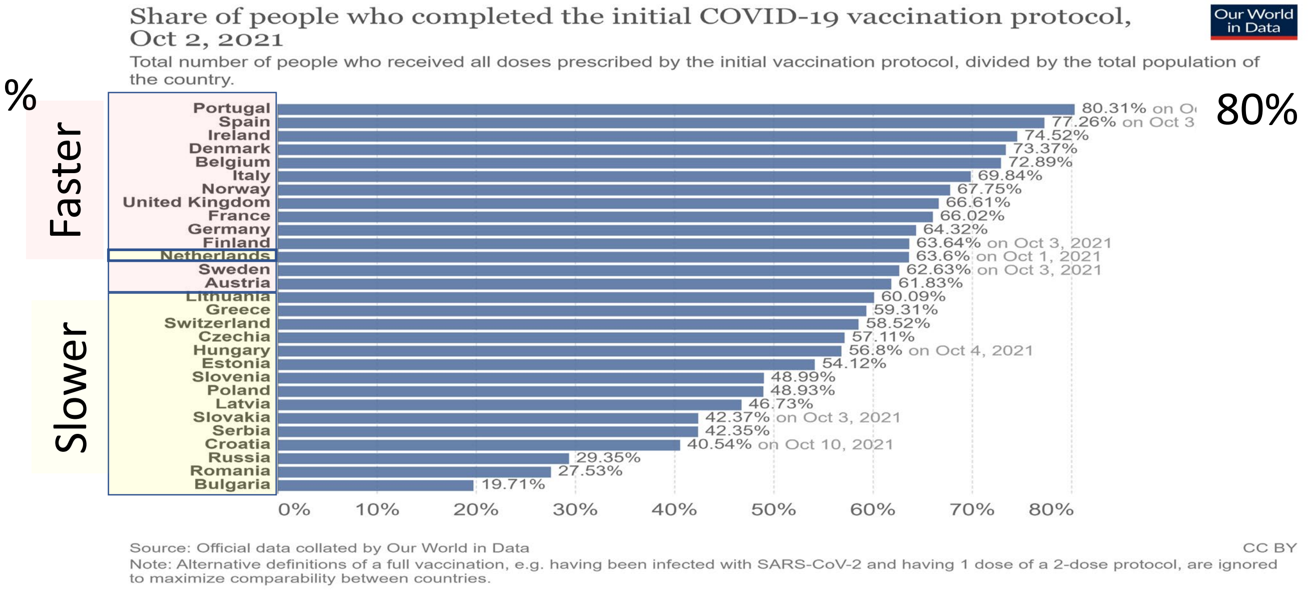

Vaccine types distribution as of May 30, 2022 in EU

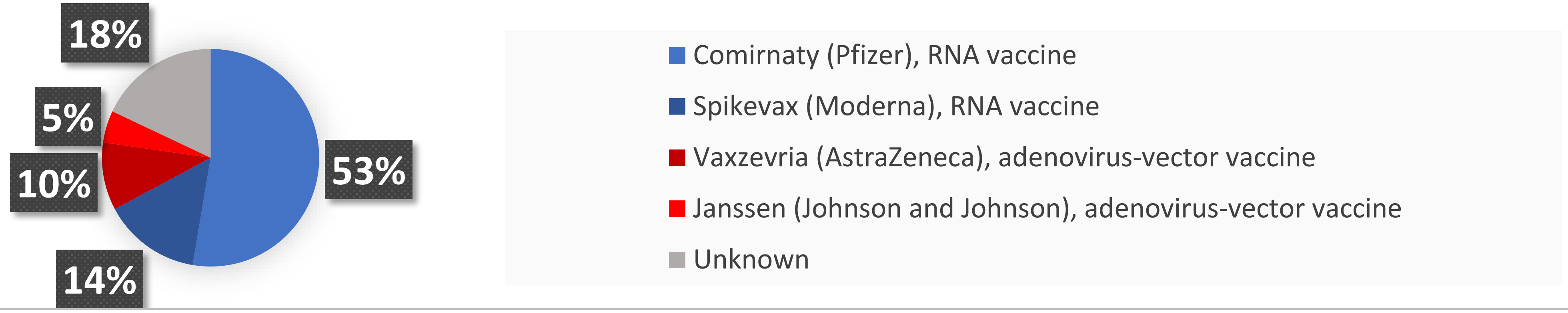

Supplement: Supplementary Figure 1 — Share of people that completed the primary series vaccination at a given date and distribution of different vaccine types in EU. [file Image_1.pdf]
