# Supplementary material for: Comparison of vaccination and booster rates and their impact on excess mortality during the COVID-19 pandemic in European countries
Source: Front Immunol. 2023 Jul 6;14:1151311. doi: 10.3389/fimmu.2023.1151311 (PMC10357837; doi:10.3389/fimmu.2023.1151311)

July 5, 2021

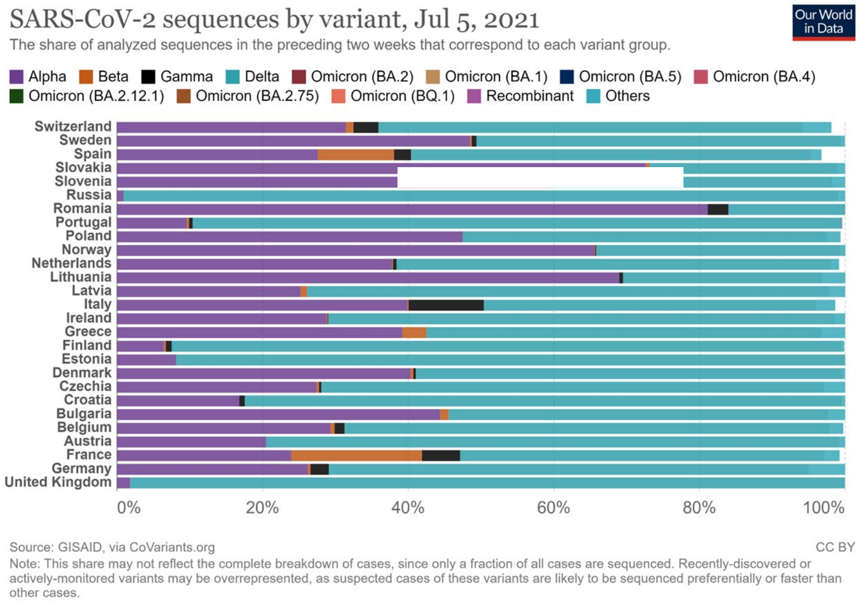

December 6, 2021

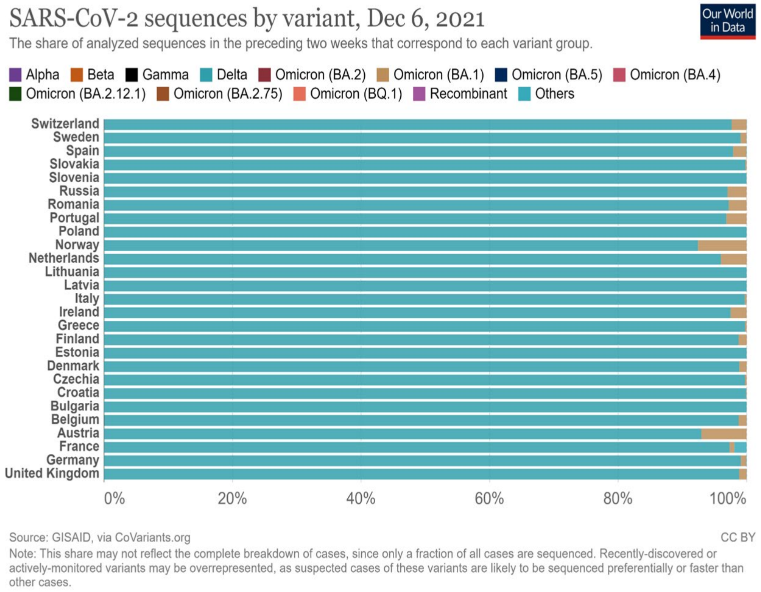

January 31, 2022

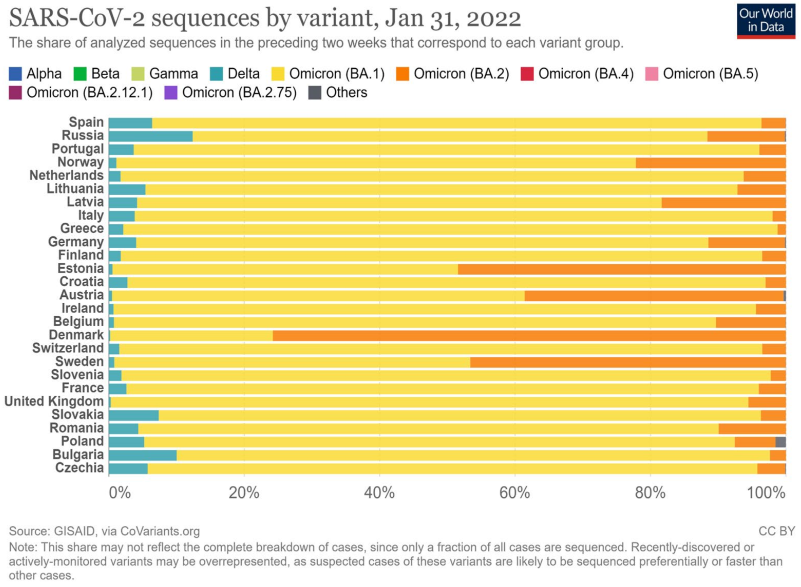

May 23, 2022

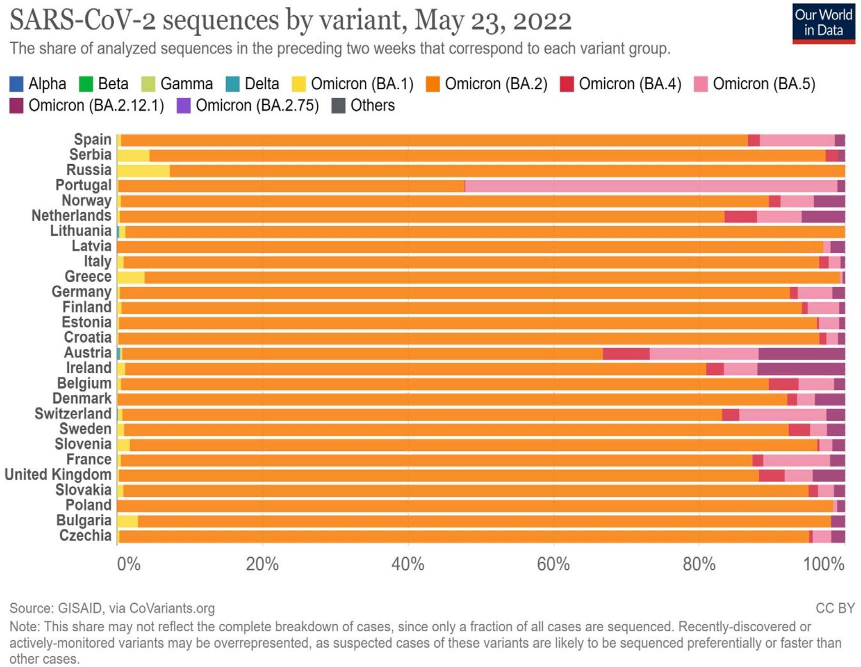

June 20, 2022

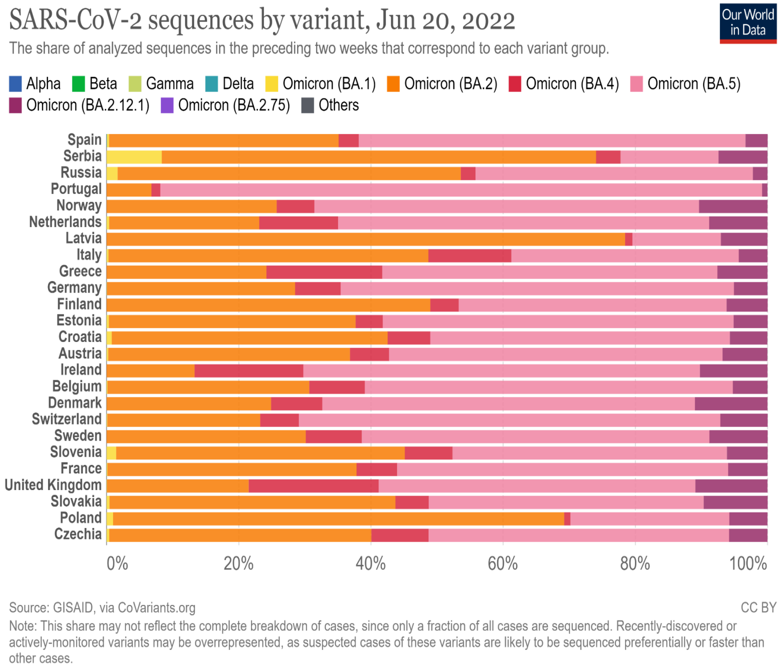

August 29, 2022

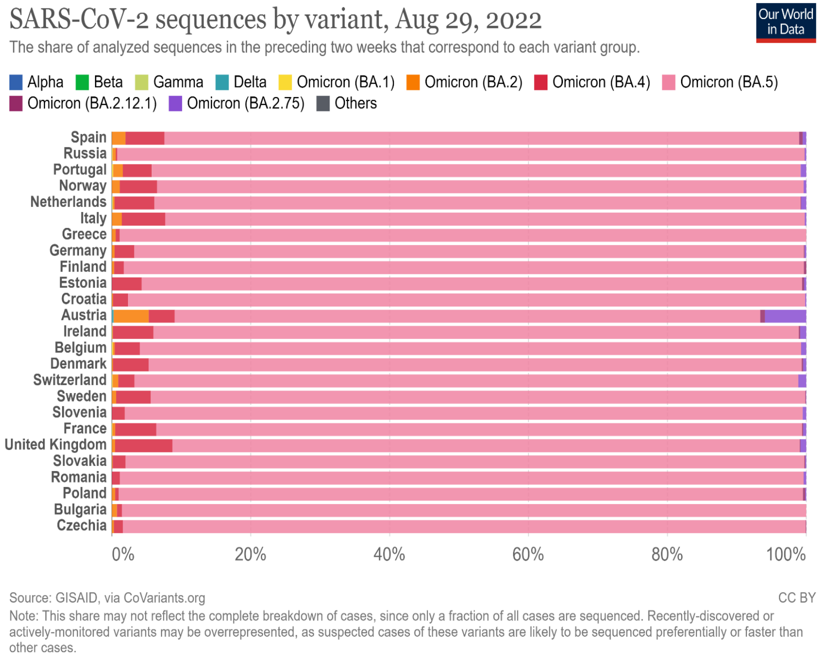

Supplement: Supplementary Figure 2 — SARS-CoV-2 sequences share by variant. [file Image_2.pdf]
