# Supplementary material for: Comparison of vaccination and booster rates and their impact on excess mortality during the COVID-19 pandemic in European countries
Source: Front Immunol. 2023 Jul 6;14:1151311. doi: 10.3389/fimmu.2023.1151311 (PMC10357837; doi:10.3389/fimmu.2023.1151311)
Supplement: Supplementary Figure 3 — Relationships between national excess mortality in countries and their age characteristics. The correlation coefficient values corresponding to statistical significance (p<0.05) are shown in white on a light brown background for the Delta period and in dark green for the Omicron period. [file Image_3.pdf]

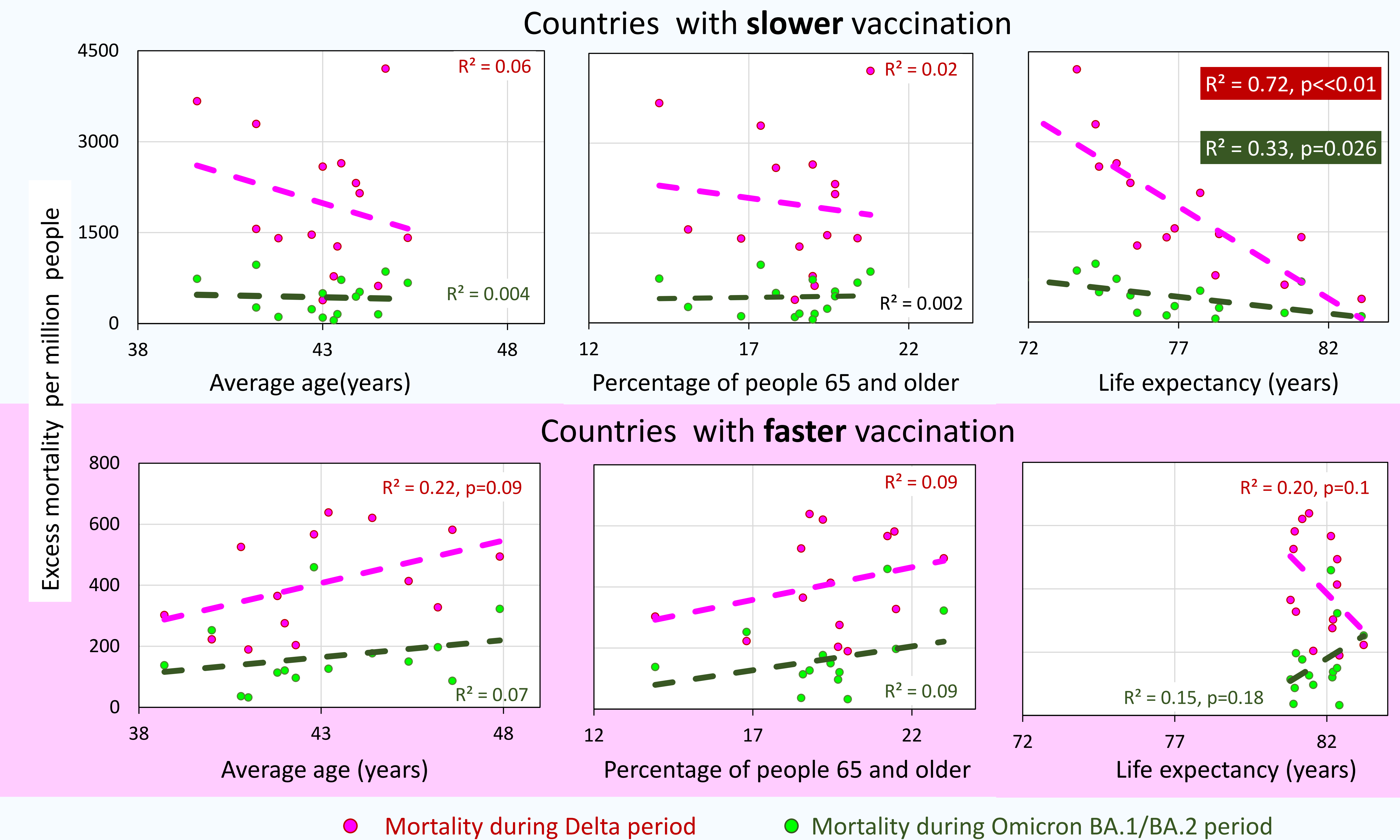
Countries with **slower** vaccination
Countries with **faster** vaccination
Excess mortality per million people
4500
3000
1500
0
800
600
400
200
0
38
43
48
12
17
22
72
77
82
Average age(years)
Percentage of people 65 and older
Life expectancy (years)
Average age (years)
R² = 0.06
R² = 0.004
R² = 0.02
R² = 0.002
R² = 0.72, p<<0.01
R² = 0.33, p=0.026
R² = 0.22, p=0.09
R² = 0.07
R² = 0.09
R² = 0.09
R² = 0.20, p=0.1
R² = 0.15, p=0.18
Mortality during Delta period
Mortality during Omicron BA.1/BA.2 period
